# Supplementary material for: Long-range charge carrier mobility in metal halide perovskite thin-films and single crystals via transient photo-conductivity
Source: Nat Commun. 2022 Jul 20;13:4201. doi: 10.1038/s41467-022-31569-w (PMC9300620; doi:10.1038/s41467-022-31569-w)
Supplement: Supplementary file 1 — Supplementary Information [file 41467_2022_31569_MOESM1_ESM.pdf]

Supplementary Information

Long-range charge carrier mobility in metal halide perovskite thin-films and single crystals via transient photo-conductivity

Jongchul Lim[1,2‡], Manuel Kober-Czerny[1‡], Yen-Hung Lin[1], James M. Ball[1], Nobuya Sakai[1], Elisabeth A. Duijnstee[1], Min Ji Hong[3], John G. Labram[3], Bernard Wenger[1]* and Henry J. Snaith[1]*

[1]Clarendon Laboratory, Department of Physics, University of Oxford, Parks Road, Oxford OX1 3PU, UK

[2]Graduate school of Energy Science and Technology, Chungnam National University, 99 Daehak-ro, Daejeon, 34134, Rep. of Korea

[3]School of Electrical Engineering and Computer Science, Oregon State University, Corvallis, OR 97331, USA

[‡] J.L. and M.K.-C. equally contributed to this work

** Correspondence:* *jclim@cnu.ac.kr*; *bernard.wenger@physics.ox.ac.uk*;

henry.snaith@physics.ox.ac.uk

Contents

- **Supplementary Fig. 1-14**
- **Supplementary Table 1-2**
- **Supplementary Note 1: The fitting of the recombination constants**
- **Supplementary References**

Supplementary Note 1: The fitting of the recombination constants

In order to take the recombination dynamics at early times during the transient photoconductivity measurements into account Supplementary Equation 1 can be used:

Supplementary Equation 1

$$\frac{dn}{dt} = G - k_1 n - k_2 n^2 - k_3 n^3$$

where n is the carrier density k_1, k_2 and k_3 are the trap-assisted, bimolecular and Auger recombination rates respectively and G is the generation rate of carriers. During pulsed excitation the generation rate will be zero, but generate an initial carrier density n_0. To estimate the recombination constants, time-resolved photoluminescence decays as well as power-dependent PLQE can be used.

In the first case, the carrier density is typically too low for k_3 to have a meaningful impact on the decays, so it can be ignored. Supplementary Equation 1 simplifies to,

Supplementary Equation 2

$$\frac{dn}{dt} = n_o - k_1 n - k_2 n^2$$

The PL from a 3D metal-halide perovskite thin-film is the result of bimolecular recombination. As such, the PL will be proportional to $k_2 n^2$.

With this, Supplementary Equation 2 can be solved based on the results from D, Lavabre et. al[1] (see Supplementary Equation 3) allowing for the estimation of k_1 and k_2 from time-resolved photoluminescence decays. All measured decays were fitted between 25 and 2000 ns with this equation using a global fitting and fixing k_1 and k_2 for all of them (see Supplementary Fig. 7):

Supplementary Equation 3

$$PL_{norm} = A \cdot k_2 \cdot \left(\frac{k_1}{(k_1 + k_2 n_0) \cdot e^{k_1 t} - k_2 n_0}\right)^2 + B$$

where PL_{norm} is the normalized PL signal k_1 and k_2 are the non-radiative and radiative recombination constants respectively, n_0 is the unattenuated carrier density and *A* and *B* are fitting parameters related to the instrument. n_0 was estimated from the laser fluence, thin-film thickness of 780 nm and an absorptance of 75%. This fitting yields a k_1 of 1.19 x 10^6 s^{-1} and a k_2 of 5.85 x 10^{-9} s^{-1} cm^3.

The decays shown in Supplementary Fig. 7 can only be fitted to Supplementary Equations 3, if the first 50 ns are ignored. This indicates that processes are more complex than Supplementary Equation 3 at early times for these samples. This

method can therefore only yield a meaningful k_1.
In fact, when fitting the transient photo-conductivity decays from Fig. 1 in the main text with Supplementary Equation 3 a good agreement can be achieved for k_1 = 1.2 x 10^6 s^{-1} (Supplementary Fig. 7). The free fits predict a k_2 of 3.95 x 10^{-10} s^{-1} cm^3.

It can be observed that the fits diverge more from the data for higher excitation densities, meaning that the bimolecular as well as Auger recombination will need to be included in the corrections.

For this reason, the recombination parameters are further refined using power-dependent PLQE, which is related to the constants via:

Supplementary Equation 4

$$PLQE = \frac{k_2 n}{k_1 + k_2 n + k_3 n^2}$$

n is the carrier density. In the case of a continuous excitation the carrier density cannot be estimated simply from the laser fluence. Instead, Supplementary Equation 1 must be solved for the case $\frac{dn}{dt}$ = 0. k_1, k_2 and k_3 must be assumed for this and where initially taken from literature. Then Supplementary Equation 4 was solved to obtain a new set of initial k_1, k_2 and k_3. This process was repeated a few times, refining the recombination constants with each iteration.
For a fixed k_1 = 1.2 x 10^6 s^{-1}, the free fit yields k_2 = 3.4 x 10^{-11} cm^3 s^{-1} and k_3 = 9.2 x 10^{-28} cm^6 s^{-1}, again for a film thickness of 780 nm and an absorptance of 75%. The measured data and resulting fit are shown in Supplementary Fig. 8.
Last, the impact of an error in k_1, k_2 and k_3 is assessed (see Supplementary Fig. 9). It becomes apparent, that k_1 and k_2 only need to be accurate within one order of magnitude for this type of thin-film to yield the same average mobility. In contrast to that, k_3 has a rather significant impact on the mobilities at higher excitation densities.
The fitting parameters used for the final mobility estimation are summarized in Supplementary Table 1:

Supplementary Table 1. Recombination parameters used for the mobility estimation in this work:

Material	k_1 (s^{-1})	k_2 (cm^3 s^{-1})	k_3 (cm^6 s^{-1})
$FA_{0.83}Cs_{0.17}Pb(I_{0.9}Br_{0.1})_3$	1.2 x 10^6	3 x 10^{-11}	1 x 10^{-27}

We note that the faster decay of the TRPL traces at higher charge carrier densities, in comparison to the TPC decay traces, is expected. It is well understood that PL intensity scales with the carrier density squared, when in the bimolecular regime, whereas conductivity will scale linearly with carrier density. Hence, as the carrier density decays after photoexcitation the PL intensity will decay as a square function of the decaying

carrier density, until the monomolecular regime in met. In contrast the TPC will decay as a linear function of the decaying carrier density. Furthermore, since the excitation source for the TRPL was 405nm wavelength, this will be predominantly absorbed near the thin film surface. The rapid diffusion of carriers away from the surface will also contribute to the early time decal of the TRPL trace. However, this diffusion is not a recombination process, and will hence not result in a drop in the TPC signal. We finally note that we employed a 470nm photoexcitation for the TPC excitation, which also has a deeper penetration depth. Additionally, during the first < 200 ns TRPL decay (fast radiative recombination as shown in Supplementary Fig. 7), our home-built TPC setup has an instrument-limited response. It highlights the strength of our post-processing on the data to accurately evaluate the mobility of the mobile charge carriers.

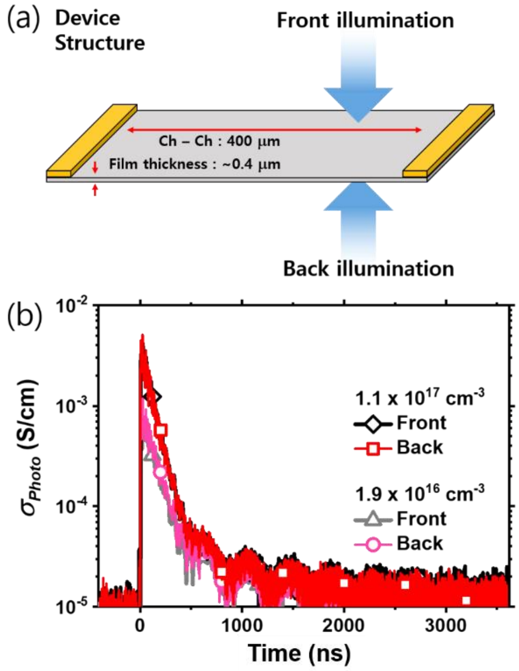

Supplementary Fig. 1. Photo-conductivity of perovskite films illuminated in different directions with different free-carrier density. (**a**) Schematic illustration of device structure showing the aspect ratio around 1000 between electrode space (400 μm) and film thickness (~ 0.4 μm), and illumination direction both front (from in-plane electrodes side) and back (from bottom side), (**b**) photo-conductivity decay profiles of FACs perovskite thin-films with different free-carrier densities (cm^{-3}), indicating the identical decay kinetics.

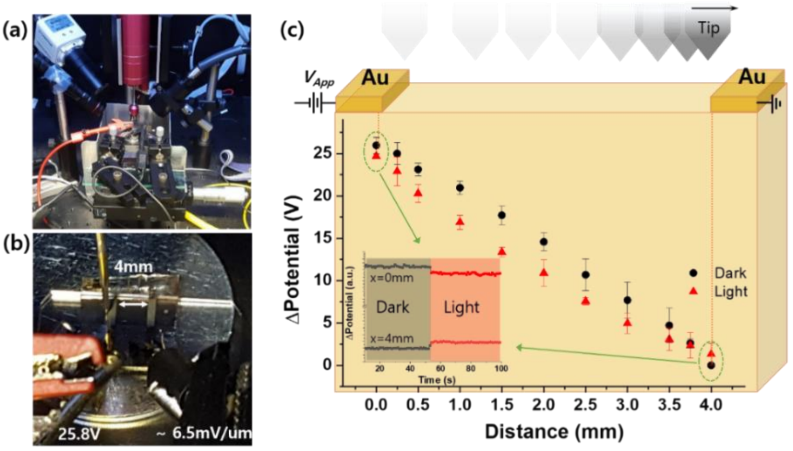

Supplementary Fig. 2. Surface potential difference for in-plane electrode device. We obtained surface potential differences of FACs perovskite films ($FA_{0.83}Cs_{0.17}Pb(I_{0.9}Br_{0.1})_3$) between in-plane electrodes under the externally applied bias voltage (< 5 mV μm^{-1}) with and without light. (**a**) Picture of experimental setup, (**b**) closer look of in-plane device and probe. (**c**) Surface potential difference measured at room temperature in nitrogen condition using a Kelvin probe (KP technologies) with a 50 μm tip diameter, and with a micro-translation stage to move the sample under the tip.

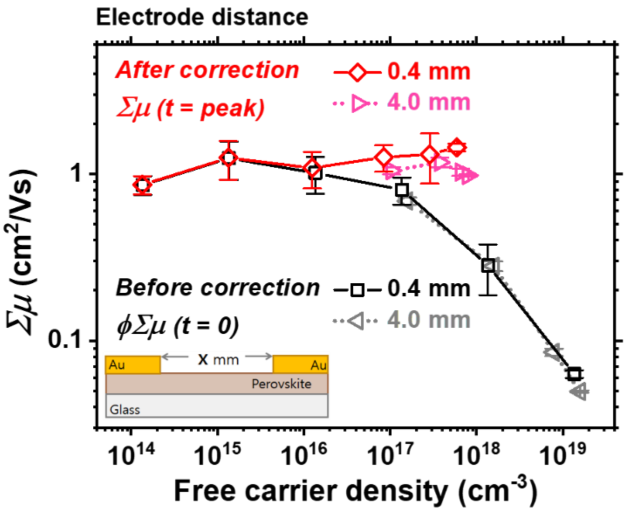

Supplementary Fig. 3. Lateral mobilities of FACs perovskite thin-films measured with different distances of in-plane electrodes. Before ($\phi\Sigma\mu_{(t=0)}$ vs. excitation density (cm^{-3})) and after ($\Sigma\mu\ (t = peak)$ vs. free carrier density (cm^{-3})) correction for 4.0 mm grey and pink lines, respectively, and for 0.4 mm black and red lines, respectively. The errors shown are statistical errors from three consecutive scans of the same sample.

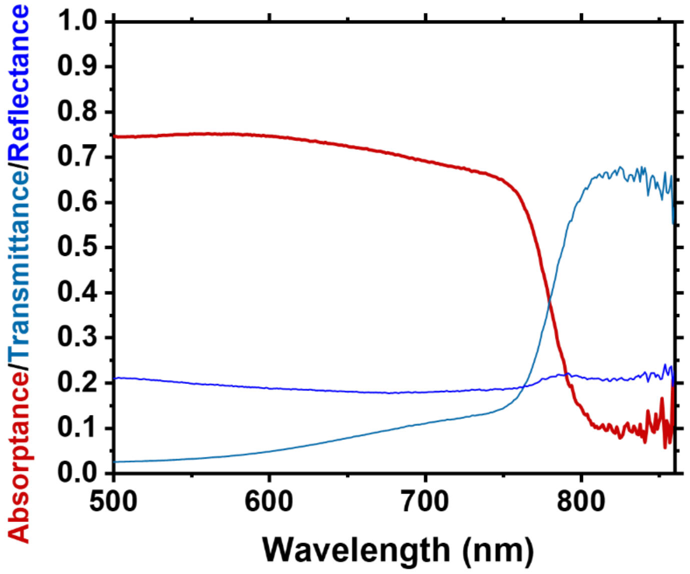

Supplementary Fig. 4. Transmittance, reflectance and absorptance of $FA_{0.83}Cs_{0.17}Pb(I_{0.9}Br_{0.1})_3$ thin-films on glass. The resulting absorptance of 75% < 500 nm was assumed for all calculations.

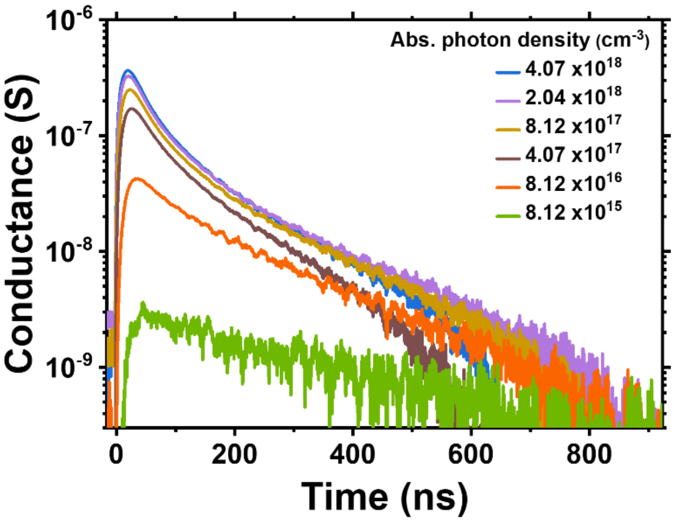

Supplementary Fig. 5. Time-resolved microwave conductivity transient data for FACs perovskite films. We obtained the change in conductance due to optical illumination as a function of time for FACs ($FA_{0.83}Cs_{0.17}Pb(I_{0.9}Br_{0.1})_3$) perovskite thin-films on quartz, at various excitation density (cm^{-3}). Samples were measured at room temperature in air.

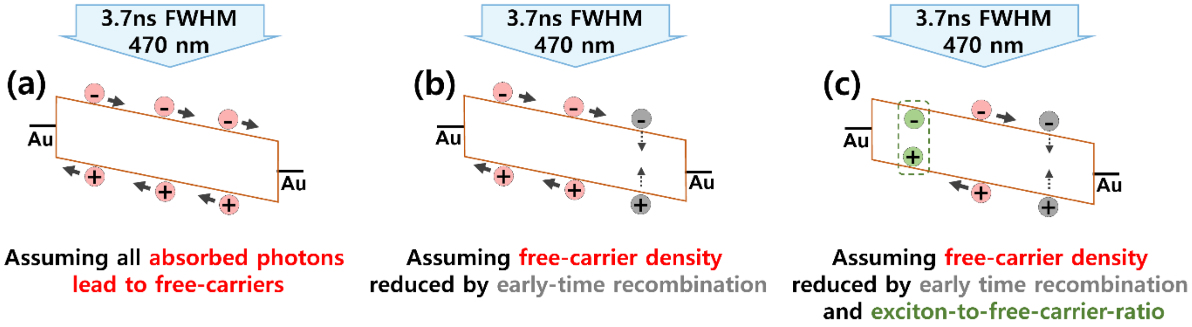

Supplementary Fig. 6. Schematic illustration of photo-induced carrier population changes within perovskite films with in-plane electrodes, (a) assuming all absorbed photons lead to free-carriers, (b) assuming free-carrier density reduced by early-time recombination, (c) assuming free-carrier density reduced by early time recombination and exciton-to-free-carrier-ratio.

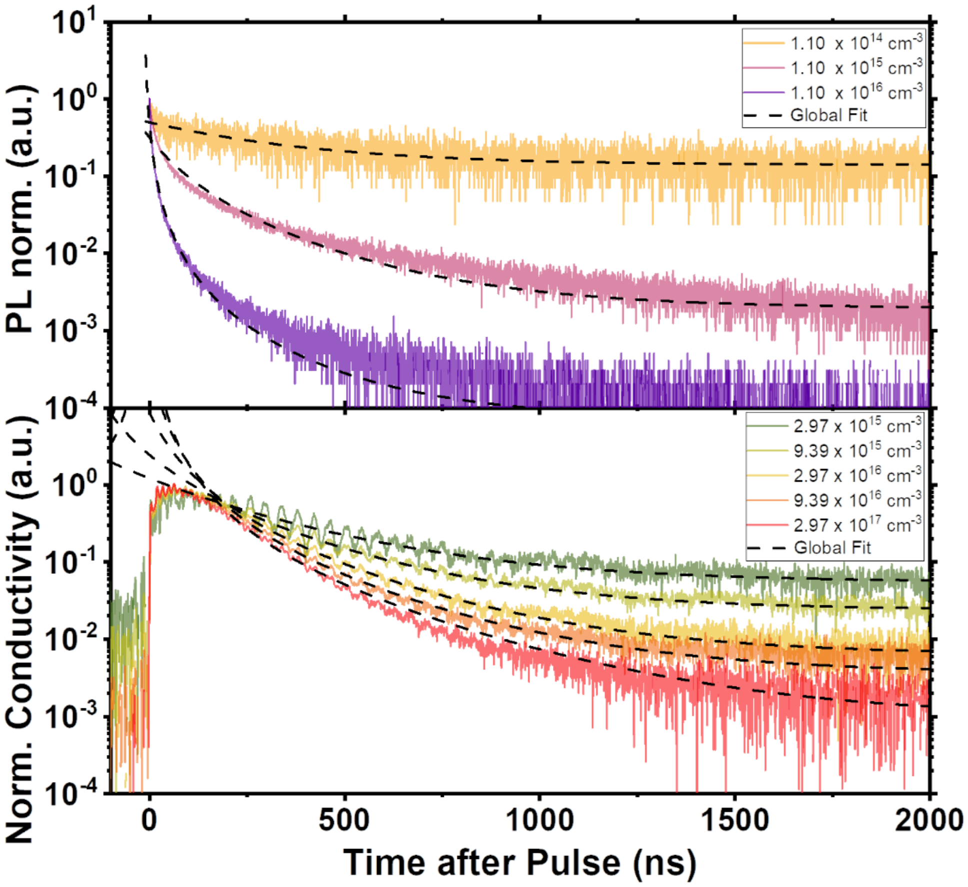

Supplementary Fig. 7. Top: Time-resolved PL decays for different fluences as well as the global fit from Equation S3 are shown. The decay is k_1-dominated. **Bottom:** Normalized transient photo-conductivity decays measured at different excitation densities and fitted with the k_1 as extracted from the TRPL decays.

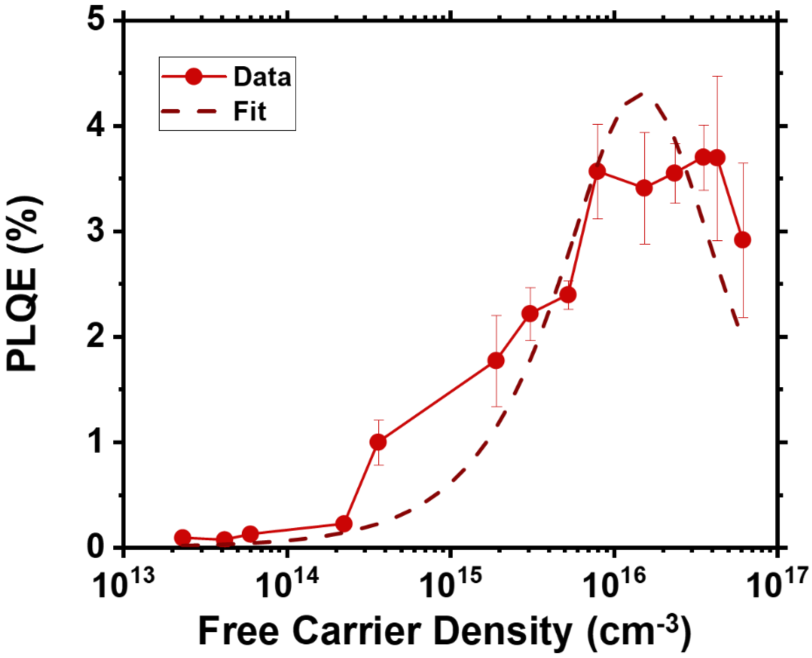

Supplementary Fig. 8. PLQE data and fitting with Equation S4 are shown. The fit corresponds to
k_1 = 1.2 x 10^6 s^{-1}, k_2 = 3.4 x 10^{-11} cm^3 s^{-1} and k_3 = 9.2 x 10^{-28} cm^6 s^{-1}.

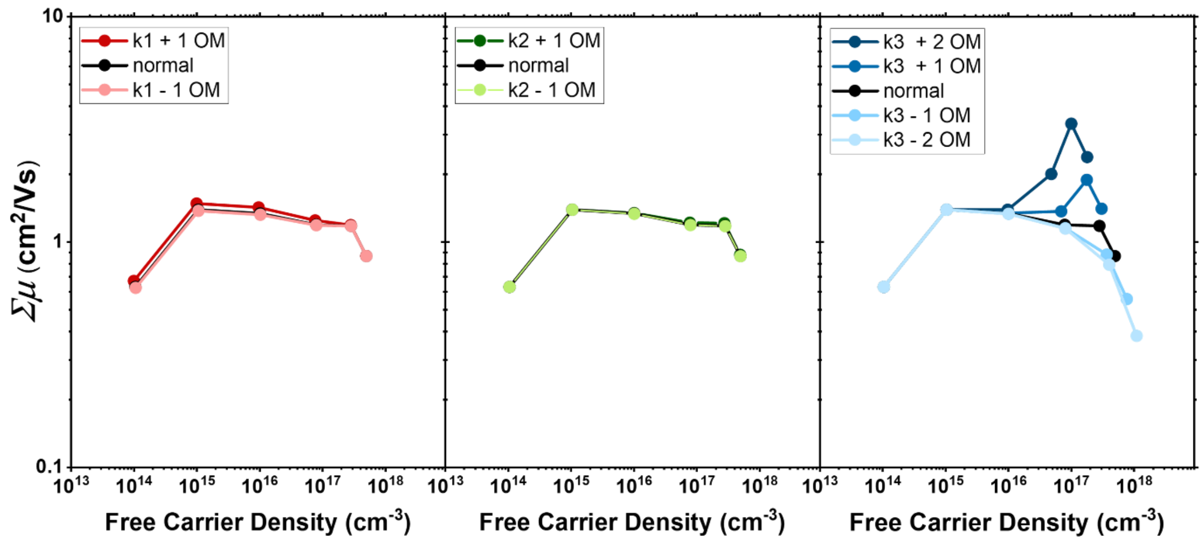

Supplementary Fig. 9. Impact of changing k_2, k_2 or k_3 by ± 1 order of magnitude (OM; or ± 2 in the case of k_3).

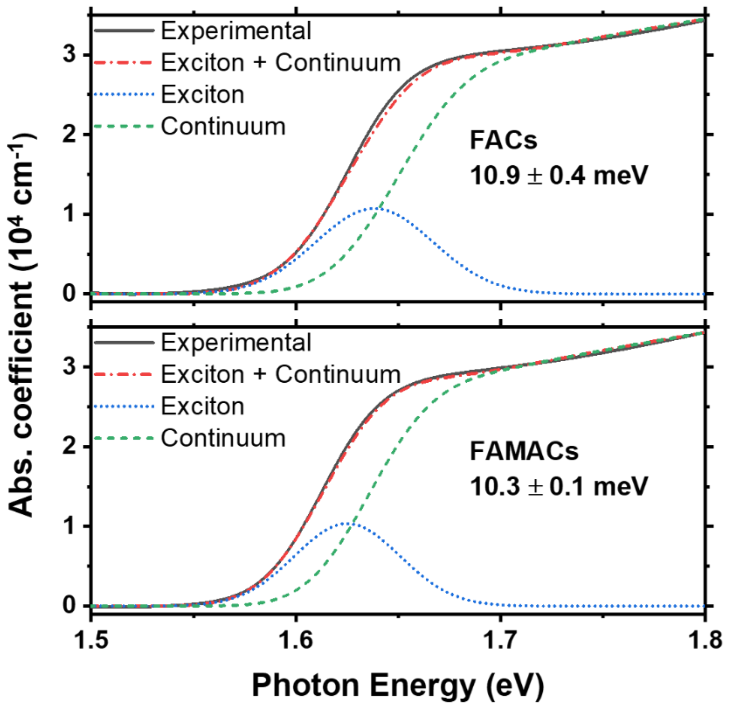

Supplementary Fig. 10. Elliot model fit of absorption profile of FACs and FAMACs perovskite thin-films for exciton binding energy (E_B) estimation.[2] From experimental data (black solid line), we obtain and plot the continuum (dashed green), exciton (dotted red) and sum of both spectra (dashed red). We determine the exciton binding energies of FACs and FAMACs to be 10.9 ± 0.4 and 10.3 ± 0.1 meV, respectively.

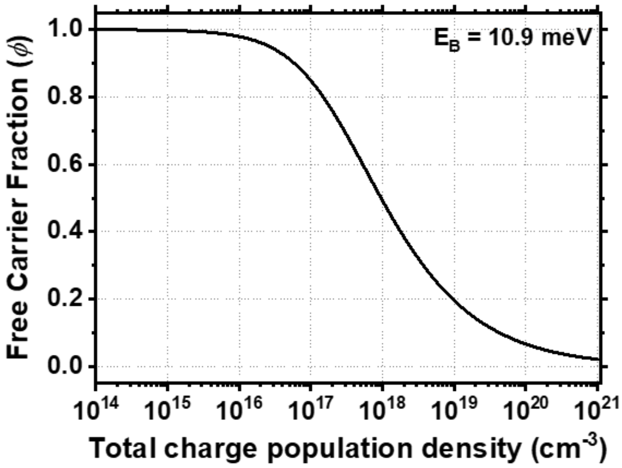

Supplementary Fig. 11. Free-carrier fraction with exciton binding energy (E_B) of 10.9 meV through Saha equation to estimate the free-carrier mobility. Here we note that total charge population density (sum of excitons and free-carriers) is equivalent to excitation density calculated with the absorption coefficient and thickness of perovskite thin-films.

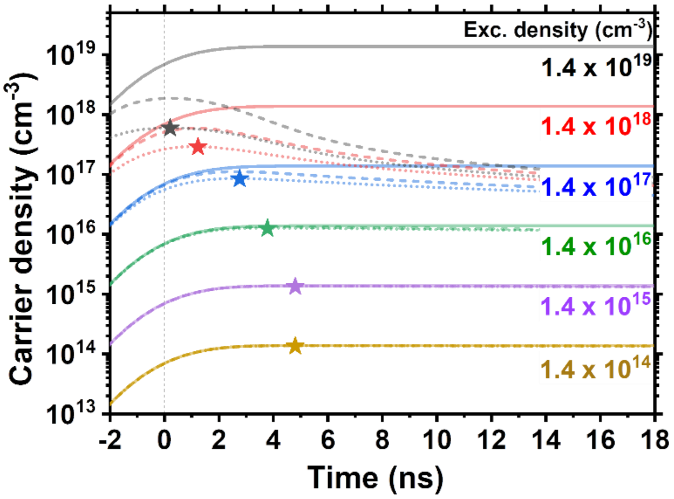

Supplementary Fig. 12. Evaluation of changes in carrier density as a function of time and the peak carrier density for FACs ($FA_{0.83}Cs_{0.17}Pb(I_{0.9}Br_{0.1})_3$) perovskite thin-films, at various excitation density (cm^{-3}). Photo-induced carrier population changes with various excitation density as a function of time with and/or without early recombination processes; excitation density (cm^{-3}) (solid line), free-carrier density (cm^{-3}) changes due to early-time recombination (dashed line), and free-carrier density (cm^{-3}) changes due to both 'early-time recombination' and 'free-carrier fraction corrected' (dotted lines), and corresponding time for peak free-carrier density (ns, closed star symbols).

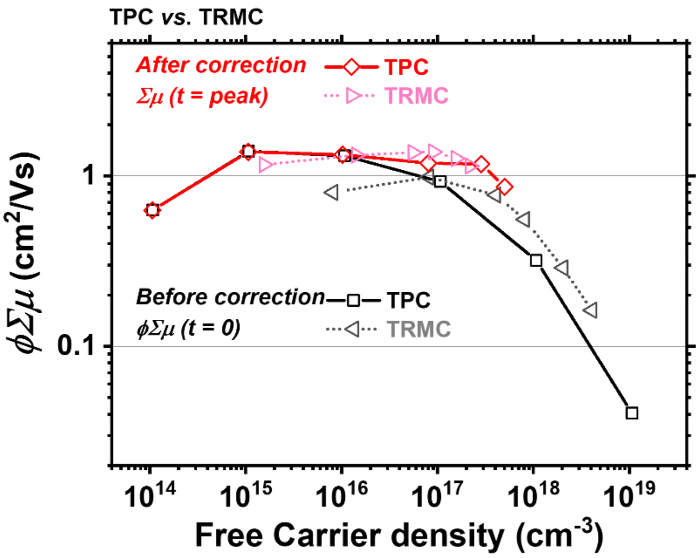

Supplementary Fig. 13. Charge carrier mobility before ($\phi\Sigma\mu_{(t=0)}$) (black square) and after ($\Sigma\mu_{(t=peak)}$) (red diamond) correction as a function of carrier density determined by TPC. Charge carrier mobility determined by TRMC before (grey left-triangle) and after (pink right-triangle) correction is in a good consistence with the mobility determined by TPC. All lines are only to guide the eyes.

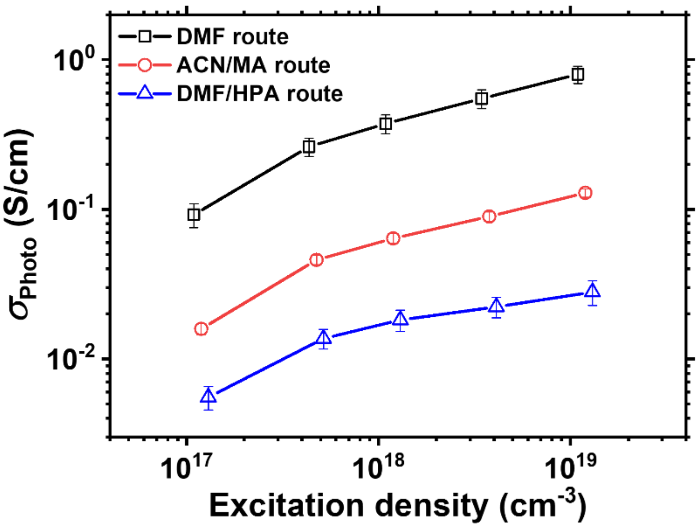

Supplementary Fig. 14. Photo-conductivity of three perovskite films prepared in different ways. Photo-conductivity values of DMF route ($MAPbI_xCl_{1-x}$), ACN/MA route ($MAPbI_3$) and DMF/HPA route ($MAPbI_3$) as a function of free carrier density (cm^{-3}).

Supplementary Table 2. Structural parameters of $MAPbI_3$ single crystal devices[a)] for TPC measurement.

Sample number	Au electrode width (mm)	Chanel to Chanel space (mm)	Crystal thickness[b)] (mm)
1	3.2	2.8	0.22 (220 μm)
2	2.8	3.3	0.30 (300 μm)
3	3.6	4.5	0.35 (350 μm)

[a)] Refer to the device architecture depicted in Fig. 4b in the main text. [b)] Crystals have around ± 5 μm of variation in thickness.

Supplementary References

1. Lavabre, D., Pimienta, V., Levy, G. & Micheau, J. C. Reversible, mixed first- and second-order and autocatalytic reactions as particular cases of a single kinetic rate law. *J. Phys. Chem.* **97**, 5321–5326 (1993).

2. Sansom, H. C. *et al.* Highly Absorbing Lead-Free Semiconductor Cu2AgBiI6for Photovoltaic Applications from the Quaternary CuI-AgI-BiI3Phase Space. *J. Am. Chem. Soc.* **143**, 3983–3992 (2021).
